# Understanding Frailty in IBD: Implications for Clinical Outcomes and Multidisciplinary Care

**DOI:** 10.3390/jcm15041440

**Published:** 2026-02-12

**Authors:** Silvia Salvatori, Irene Marafini, Giovanni Monteleone

**Affiliations:** 1 Department of Systems Medicine, University of Rome “Tor Vergata”, 00133 Rome, Italy; silvia.salvatori@ptvonline.it (S.S.); irene.marafini@ptvonline.it (I.M.); 2 Gastroenterology Unit, Fondazione Policlinico “Tor Vergata”, 00133 Rome, Italy

**Keywords:** frailty, crohn disease, ulcerative colitis

## Abstract

Frailty is a complex syndrome characterized by a gradual decline in physical ability and a higher sensitivity to external stress. While it has traditionally been linked to aging, frailty is increasingly seen as an important factor affecting the health of patients with inflammatory bowel disease (IBD). IBD mainly affects the gastrointestinal tract but is often associated with systemic manifestations, such as extra-intestinal symptoms/signs, malnutrition, muscle loss, and other health problems, all of which could contribute to frailty. There are various tools to assess frailty in IBD patients, even though it is often not recognized or evaluated well in standard clinical practice. Recent evidence indicates that frailty in IBD may be partly driven by ongoing inflammation and is an independent predictor of negative health outcomes (e.g., hospitalization rates, surgical complications, and mortality). Proper management of frailty in IBD needs a broad, team-based approach that focuses on controlling disease symptoms, improving nutrition, physical abilities, and managing other comorbidities. This review aims to give a clear overview of the causes, clinical evaluation, and treatment options for frailty in IBD, emphasizing the need for early detection and intervention.

## 1. Introduction

Inflammatory bowel disease (IBD), which encompasses Crohn’s disease (CD) and ulcerative colitis (UC), is a chronic, relapsing inflammatory disorder that primarily affects the gastrointestinal tract but can also have systemic effects [1]. IBD affects millions of people of all ages, significantly impacting both older and younger groups [2]. Although IBD has long been considered a disease predominantly affecting high-income countries, recent decades have seen a rapid increase in both incidence and prevalence in newly industrialized countries, with Asia and India accounting for the highest number of new IBD diagnoses [3,4].

The cause of IBD is still unknown. However, in recent decades, several epidemiological and molecular studies have contributed to advancing our understanding of the mechanisms driving the pathological process in both CD and UC [5]. Various environmental factors (e.g., diet, infections, smoking, and stress) interact with genetic susceptibility and the immune system to shape the disease course [6,7,8,9,10,11,12,13]. Additionally, a growing body of research suggests that the intestinal microbiome plays a pivotal role in driving an exaggerated immune–inflammatory response in the gut, contributing to the initiation and perpetuation of disease [14,15,16]. At the molecular level, the aberrant activation of both innate and adaptive immune cells results in enhanced production of effector cytokines and chemokines, which contribute to amplifying the destructive inflammatory reaction [7,17]. Consistently, innovative therapies targeting specific immune pathways have been developed, thus expanding the therapeutic options available for patients. Biologic agents, such as anti-TNF therapies and IL-12/23 inhibitors, along with small-molecule inhibitors, have contributed to improve the management of IBD patients. Nonetheless, a substantial number of patients continue to experience persistent disease activity, frequent hospitalizations, and progressive disability [18,19].

Frailty is a complex condition marked by reduced physical ability and greater vulnerability to negative outcomes after stressors (e.g., infections, surgery) [20]. In IBD, frailty may result from several factors, including chronic mucosal inflammation, malnutrition, loss of muscle mass, and the cumulative impact of other comorbidities [21,22,23]. Importantly, frailty is not limited to older patients, but can occur in younger individuals, and preliminary observations suggest that frail IBD patients could have a higher risk of negative clinical outcomes [24]. Notably, patients with IBD show a higher risk of being frail [25,26]. This may be due to the systemic involvement of the disease itself, particularly during active phases, leading to clinical symptoms that often overlap.

This review aims to explore the prevalence and pathophysiology of frailty in IBD, the diagnostic tools, and the strategies for managing the frail patients. We performed a literature search to explore the relationship between IBD and frailty, focusing on the most relevant articles and reviews published in recent years.

## 2. Prevalence and Pathophysiology of Frailty in IBD

The prevalence of frailty in patients with IBD varies depending on the study population, patient characteristics, and diagnostic methods. In a cohort of 11,001 IBD patients, Kochar et al. found a frailty prevalence of 6%. Frailty rates increased with age, rising from 4% in patients aged 20 to 29 years to about 25% in those over 90 years. Frail patients had a higher risk of death, regardless of confounding factors such as age, sex, disease duration, comorbidities, IBD-related surgery, and immunosuppressive therapy [27]. In a larger study of 47,402 hospitalized IBD patients, Qian et al. reported a frailty prevalence of 32.7%. Frailty was associated with higher mortality, readmission rates, and longer hospital stays [28]. A study using ICD-9-CM codes for frailty in hospitalized IBD patients found an 11% prevalence, with frailty linked to increased mortality and readmission rates over five-years [29]. In a Chinese cohort, 14% of patients were frail, with nearly 60% labeled as “prefrail”. Women were more likely to be frail, and depression was a significant risk factor [30]. An Italian cohort study involving 386 outpatients found 17% of IBD patients met frailty criteria [26], with some experiencing reversibility of the frailty status [31]. A systematic review and meta-analysis of seven retrospective and two prospective cohort studies, encompassing 1.5 million IBD patients, found a combined frailty prevalence of 18%: frailty was associated with higher mortality and readmissions, and frail patients were less likely to have IBD-related surgery [32]. A similar prevalence (18%) was reported in a meta-analysis of 23 observational studies with 1.9 million adults, where frailty was linked to higher post-operative complications and infection-related hospitalizations [24].

These findings indicate that nearly one-fifth of IBD patients are frail. As expected, frailty is more common in older IBD patients, though younger individuals with severe or poorly controlled disease may also be affected. Although it remains unclear whether pediatric patients with IBD can be classified as frail, and which assessment tools would be most appropriate in this population, growth retardation—primarily driven by chronically active disease and prolonged steroid therapy—may increase the risk of developing frailty later in life. However, further evidence is needed to support this hypothesis. The development of an IBD-specific frailty assessment score could improve the identification and evaluation of frailty, including in younger patients.

Notably, the close relationship between active IBD and frailty deserves particular attention. Patients with active disease are more likely to experience weight loss, varying degrees of malnutrition, and loss of muscle mass, as well as to require multiple therapies, particularly corticosteroids. In cases of severe disease activity, the risk of hospitalization is also increased. Collectively, these factors contribute to a higher risk of frailty.

Despite the identification of several risk factors for frailty, there is a variability in frailty prevalence reported across different studies that may be explained by differences in study design, population characteristics, and the frailty assessment tools employed.

**Figure 1:**
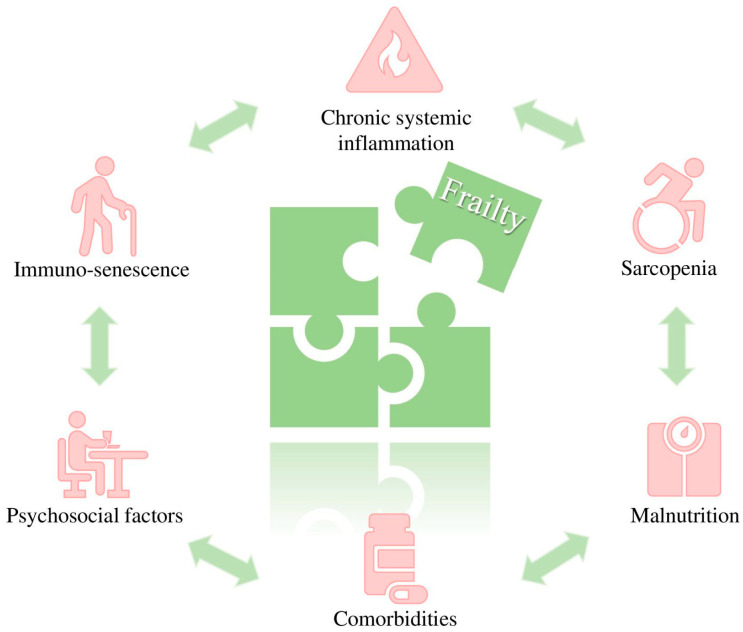
Interconnected factors underlying the pathophysiology of frailty in IBD.

The pathogenesis of frailty in IBD is complex and involves inflammatory, metabolic, and psychosocial factors. Chronic systemic inflammation seems to be a primary factor, with pro-inflammatory cytokines [e.g., tumor necrosis factor-alpha (TNF-α), interleukin-6 (IL-6)] promoting muscle catabolism, energy depletion, and impairment of the reparative processes. Over time, chronic inflammation contributes to immune aging, increasing the risk of infections and worsening frailty [33,34]. Research by Asscher et al. showed that both clinical and biological disease activity in IBD was related to deficits in geriatric assessments, suggesting a strong link between disease activity and frailty [35]. Similarly, Kochar et al. demonstrated that treating elderly IBD patients with anti-TNF drugs improved frailty status [36]. Our data also indicated that disease activity was an independent risk factor for a frail phenotype, and resolution of the active disease was associated with frailty reversibility only in some subsets of patients [26,31], suggesting that additional factors other than ongoing inflammation contribute to frailty in IBD. One such factor could be sarcopenia, or the loss of muscle mass and function. A systematic review of 35 studies reported a 17% prevalence of sarcopenia in IBD patients [37]. Factors contributing to this include malnutrition, physical inactivity due to fatigue and gastrointestinal symptoms, chronic inflammation, and the use of corticosteroids [38]. Comorbidities such as heart disease, diabetes, osteoporosis, and extraintestinal manifestations lower physiological reserves and increase vulnerability to stress [39]. Psychosocial factors like depression, anxiety, and social isolation also contribute significantly to frailty. The emotional burden of chronic disease can make self-care and treatment adherence more challenging, leading to functional decline. A multicenter prospective study found that nearly one-third of newly diagnosed IBD patients faced anxiety and depression, which negatively affected their quality of life [40] (Figure 1).

Taken together, these factors highlight the multifactorial nature of frailty in IBD, although the relative contribution of each factor to an individual’s frailty status remains unclear. Given the significant impact of frailty on quality of life and clinical outcomes in patients with IBD, several biochemical markers have been proposed to improve our understanding of this condition and to enable earlier identification. Inflammatory markers, including circulating serum levels of interleukin (IL)-6, tumor necrosis factor receptors (TNFR)-1 and -2, and C-reactive protein (CRP), as well as clinical, metabolic, and genetic markers, have been investigated. However, the available evidence in IBD remains limited. As a result of this paucity and heterogeneity of data, none of these biomarkers is currently used in routine clinical practice, and further well-designed studies are needed to clarify their role [26,41,42].

Alongside radiographic and ultrasound-based skeletal and muscle biomarkers, novel approaches have also been proposed, including brain MRI, which can assess specific brain changes associated with frailty, such as increased total white matter hyperintensity burden and reduced gray matter volume [42]. However, data on these latter technologies in IBD are still lacking.

## 3. Frailty Assessment Tools in IBD

In IBD, the frailty status can be evaluated using several tools, each with distinct strengths and limitations (Table 1).

The Hospital Frailty Risk Score (HFRS) uses 109 specific ICD-10 codes to group patients into low, intermediate, or high risk of frailty [43]. Because it is based on electronic records, its applicability is favored in hospitalized patients but not in outpatients. The Fried Frailty Phenotype [44], one of the most widely used tools in the IBD literature, defines frailty using five physical parameters: unintentional weight loss, exhaustion, reduced grip strength, slow gait speed, and low physical activity. Meeting three or more criteria indicates frailty, while one or two criteria identify the pre-frail status. It focuses primarily on physical performance and does not take into consideration cognitive or psychosocial components. The Clinical Frailty Scale [20] (CFS) offers a rapid, intuitive tool based on functional status, comorbidities, and overall fitness. While practical, it may miss frailty in younger people or those with milder symptoms. Another option is the Frailty Index [45], which incorporates comorbidities, laboratory values, cognitive decline, and functional deficits to generate a continuous measure of frailty. Unfortunately, this tool needs extensive data, and therefore, its routine use is limited.

Additional tools can be used to integrate the clinical evaluation of frailty. For example, the G8 questionnaire, originally designed for geriatric patients with malignancies, can be used to assess nutritional status, mobility, neuropsychological issues, polypharmacy, and general health. In IBD, it can help identify geriatric deficits among older adults [46]. Patient-reported outcome measures, including the Short Form-36 and IBD-specific quality-of-life tools, are useful to identify subjective elements of the frailty status, such as fatigue, functional capacity, and psychosocial well-being, but they may be influenced by mood disorders or active disease [47].

Cross-sectional imaging allows the quantification of skeletal muscle mass and the diagnosis of sarcopenia [48]. Emerging data support the use of muscle ultrasound as a non-invasive method for diagnosing sarcopenia. In a recent prospective study of 153 IBD patients, ultrasound of muscle thickness demonstrated comparable performance to bioelectrical impedance analysis and magnetic resonance imaging, with a reported sarcopenia prevalence of 50% [49].

The current evidence underscores the importance of a multidimensional assessment strategy that integrates physical, nutritional, cognitive, and psychosocial domains to carefully ascertain the frailty status.

The large number of existing tools for evaluating frailty in IBD patients, coupled with the lack of comparative studies, currently prevents any recommendations for their use in specific clinical settings. Some tools are designed specifically for outpatient settings, while others are tailored for hospitalized patients, as shown in Table 1. This distinction is one of the few factors clearly differentiating their applicability. To establish a practical approach for frailty assessment in clinical practice, it would be valuable to either standardize the existing tools or develop a new one specifically designed and validated for the IBD population [50]. Emerging technologies, such as digital health solutions and biomarkers, could further enhance the early detection of frailty, supporting its routine evaluation in clinical practice.

## 4. Clinical Implications of Frailty in IBD

**Figure 2:**
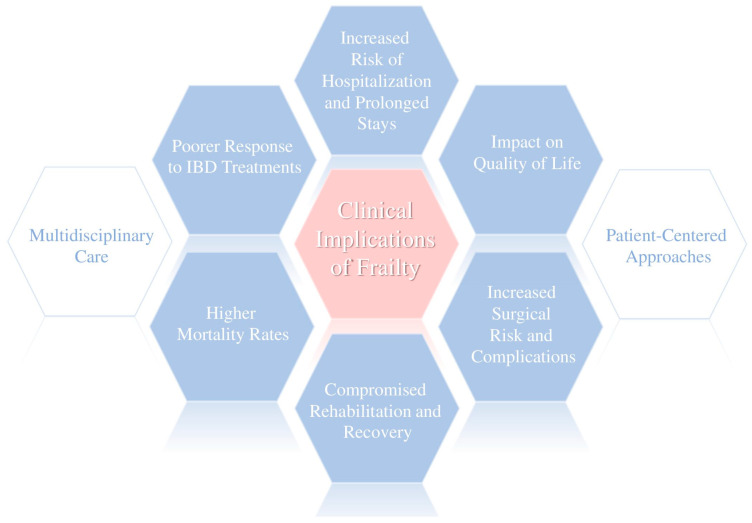
Clinical implications of frailty in IBD.

Frailty in people with IBD brings numerous challenges, not just medically, but also in terms of surgery and overall quality of life. Clinically, it represents an important marker of biological vulnerability, which could help identify patients at a higher risk for adverse health outcomes (Figure 2).

Frail patients are more likely to be hospitalized, tend to stay in the hospital longer, and have a higher chance of being readmitted after discharge. In a cohort study of 405 older IBD patients, Asscher and colleagues showed that frailty was independently associated with a higher likelihood of acute hospitalization (aHR 2.2, 95% CI 1.3–3.8), as well as significant declines in quality of life (aOR 2.1, 95% CI 1.3–3.6) and functional status (aOR 3.7, 95% CI 1.7–8.1) [47]. Qian et al. reported that frailty in hospitalized IBD patients was associated with higher rates of readmission, both for IBD-related complications and for other medical causes, thus increasing healthcare costs [28].

Frailty can influence drug metabolism and pharmacokinetics, potentially altering the efficacy and safety of drugs. Reduced hepatic and renal function, which are common in frail individuals, may impair drug clearance and increase the risk of toxicity. Support to this comes from a retrospective study of 366 IBD patients documenting that nearly two-thirds of older patients had significant comorbidities, and those with comorbid conditions were more likely to discontinue biologic therapy due to hypersensitivity reactions and abnormalities in liver enzymes [51]. Unfortunately, elderly patients are often excluded from clinical trials, limiting the ability to evaluate potential strategies, such as dose reduction or alternative dosing schedules, to minimize these side effects.

Frailty has a substantial impact on surgical outcomes in IBD patients. Evidence indicates that frail individuals undergoing procedures such as bowel resection, colectomy, or ileal pouch-anal anastomosis have a higher risk of postoperative complications (i.e., wound infections, anastomotic leaks, delayed healing, and prolonged recovery). Additionally, frailty is associated with increased short-term postoperative mortality. Telemi and colleagues retrospectively analyzed 943 UC patients undergoing colectomy and found that frailty was independently associated with septic complications (aOR 31.26, *p* = 0.006), cardiopulmonary complications (aOR 216.3, *p* ≤ 0.001), serious morbidity (aOR 66.8, *p* ≤ 0.001), overall morbidity (aOR 25.5, *p* ≤ 0.001), Clavien class IV complications (aOR 204.9, *p* ≤ 0.001), and need for reoperation (aOR 14.29, *p* = 0.048) [52]. In a study of 9023 CD patients undergoing intestinal resection, Wolf and colleagues demonstrated that frailty is a stronger predictor of postoperative morbidity than chronological age [53]. These findings underscore the importance of preoperative frailty assessment in surgical candidates. Identifying frailty can guide targeted interventions, such as nutritional optimization, physical conditioning, and psychosocial support, thereby reducing complication risks and improving postoperative outcomes.

Frailty has been associated with lower rates of remission and mucosal healing, particularly in patients with aggressive or frequently relapsing disease [54,55]. Additionally, frailty increases the risk of infections and impaired wound healing in patients receiving anti-TNF agents or immunomodulators. Kochar and colleagues reported that frail patients treated with these therapies had a significantly higher risk of infection compared to fit patients, independent of age, comorbidities, or concomitant medications [56].

Although frailty is not exclusively a pathological condition affecting older adults with IBD, they have an increased likelihood of being frail, which is associated with a higher risk of mortality and hospital admission [53]. Notably, patients with CD have a higher risk of frailty compared to those with UC [27,35].

Frailty in IBD can also increase the risk of depression, anxiety, and social isolation, which in turn impairs quality of life and disease self-management [57,58]. Cognitive deficits, such as impaired memory and concentration, may reduce medication adherence and hinder self-care. Social isolation, often worsened by the physical and emotional burdens of frailty, further diminishes resilience and coping capacity [59]. Together, these psychosocial factors compromise engagement with healthcare providers, hinder functional recovery, and negatively impact disease outcomes [60]. Given the profound impact of psychological well-being on IBD activity, and conversely the influence of disease activity on frailty, it is of primary importance to incorporate psychological support for all IBD patients into routine clinical practice [61].

## 5. Management of Frailty in IBD

The management of frailty in IBD requires a holistic, multidisciplinary approach, in which gastroenterologists, dietitians, physiotherapists, psychologists, and primary care providers collaborate to offer individualized therapies [62]. Effective management of frailty combines treatments that address the underlying disease, supportive care, and healthy lifestyle changes, to preserve functional status, prevent complications, and improve the overall quality of life (Table 2) [63].

The priority is managing IBD activity, as ongoing inflammation is a major driver of frailty at least in some subsets of individuals. Biologic therapies and small molecules should be tailored to disease severity to control the detrimental immune–inflammatory response and inhibit the production/function of cytokines (e.g., TNF-α), which contribute to muscle catabolism, fatigue, and frailty progression. Steroid-sparing strategies are also crucial in frail patients due to the iatrogenic effects of corticosteroids [58].

Nutritional interventions are equally relevant since malnutrition plays a central role in frailty. Nutritional assessment, using tools like the Mini Nutritional Assessment or Subjective Global Assessment, can help identify at-risk patients [64]. A protein-rich diet, oral supplements, and micronutrient support (e.g., vitamin D, B12, iron, zinc) are essential to maintain muscle mass, immune function, and overall recovery. In cases of severe malabsorption or during acute disease flares, enteral nutrition may be required, with parenteral nutrition reserved for refractory disease or post-surgical complications [65].

Physical rehabilitation is pivotal in addressing sarcopenia and preserving functional capacity. Exercise programs, and especially those that include resistance training, have been shown to boost muscle strength and overall physical performance [66]. In addition, physical therapy that focuses on improving balance and preventing falls can help increase independence and quality of life [67].

Because frail people with IBD often have other health conditions like heart disease, osteoporosis, or diabetes [68], it is crucial to actively manage these conditions, by adjusting medications, encouraging healthy lifestyle changes, and regular monitoring to prevent further health deterioration.

Supporting mental and emotional health is another crucial part of managing frailty as already underlined above. It is important to regularly screen for depression and anxiety and to provide the appropriate interventions (e.g., cognitive–behavioral therapy, stress management, antidepressants) when needed. Building social support networks through patient groups or caregiver involvement helps reduce social isolation and ensures patients feel supported throughout their care [69].

For patients requiring surgery, rehabilitation programs focused on nutrition, physical conditioning, and optimizing comorbidities are essential to minimize postoperative complications [70]. Preoperative frailty assessments help stratify surgical risk and optimize perioperative care, including early mobilization, wound management, and postoperative monitoring [4,52,53].

Considering the importance of this condition and its consequences in patients with IBD, frailty should be assessed as early as possible and monitored over time, even though the impact of such assessment on clinical outcomes is not yet established, nor is the most appropriate method among the available scoring tools. However, implementing all of the proposed strategies in daily clinical practice is often challenging, primarily due to limited financial resources, time constraints, adherence issues, and variability in access to multidisciplinary teams.

## 6. Conclusions

Frailty in IBD is becoming more widely recognized as a significant condition that affects both the clinical outcomes and quality of life of patients. As the global prevalence of IBD rises, understanding the factors contributing to frailty in these patients becomes essential. Frailty is linked to adverse outcomes, such as increased hospitalization, poor treatment response, and an increased risk of infections, highlighting the need for accurate diagnosis and management [24]. A multidisciplinary, patient-centered approach that aims at the early detection and personalization of interventions is crucial for reducing the adverse consequences of this condition in IBD patients. While useful, the existing screening tools are not always effective in identifying frailty, particularly in young individuals or patients without obvious physical signs. Therefore, future research is needed to improve such tools, possibly incorporating biomarkers, advanced imaging techniques, and digital health technology.

Although current IBD therapies are effective in controlling the pathogenic inflammation and improving clinical outcomes in most patients, their impact on frailty remains underexplored. Since certain aspects of frailty are linked to IBD activity and may be reversible, inducing mucosal healing could potentially alleviate frailty [26,36]. However, it is still unclear how different treatment regimens affect frailty, particularly in terms of muscle mass, nutrition, and physical function. There is a need for more longitudinal studies to understand the long-term effects of frailty on the progression of IBD. Investigating whether frailty accelerates complications such as cancer or fibrosis in IBD patients would be valuable. Additionally, examining whether addressing frailty improves long-term outcomes, such as reducing the need for surgical interventions, will be essential in establishing frailty as a key prognostic marker in IBD.

After discussing the main factors contributing to frailty and the principal strategies to assess and potentially mitigate this condition, it is evident that current research faces several challenges. To date, frailty in IBD patients has largely been evaluated using assessment tools validated in general geriatric populations, which may introduce bias in estimating the true prevalence of this condition in this population. In addition, most existing studies are retrospective and show considerable heterogeneity in both the tools used and the characteristics of the study populations. Future research should focus on developing an assessment tool specifically designed for detecting frailty in IBD patients, in order to clarify the true impact and burden of this condition. Finally, older adults and frailty assessment should be incorporated into clinical interventional trials, to enhance our ability to manage advanced therapies in the patients at greatest risk.

## Figures and Tables

**Table 1:** Most commonly used frailty assessment tools in IBD.

Frailty Assessment Tool	Thresholds	Strengths	Weaknesses
Hospital Frailty Risk Score (HFRS) [43]	high frailty risk: >15	Easily integrated into hospital electronic systems, objectivity	Limited applicability in outpatient settings
The Fried Frailty Phenotype [44]	≥3 criteria	Applicability in the outpatient setting	Focused on physical performance without cognitive or psychosocial assessment
Clinical Frailty Scale (CFS) [20]	7-point scale from 1-fit to 7-severely frail	Applicability in the outpatient setting	Low sensitivity for younger adults or to detect more subtle functional limitations
Frailty Index (FI) [45]	Quantifies frailty by counting the proportion of an individual’s health “deficits” compared to the total number of deficits considered.	Nuanced risk stratification	Requires extensive data, restricting its routine use
G8 questionnaire [35]	Positive screen: ≤14	Short and simple but developed as a screening tool for identifying which oncology patients required more in-depth geriatric assessment, not intended to diagnose frailty	Developed as a screening tools for geriatric deficits not for assessing frailty

**Table 2:** Main strategies for managing frailty in patients with IBD.

Management of Frailty in IBD	Key Strategies
Optimizing Disease Control	Conventional and/or advanced therapies Minimizing corticosteroid exposure
Nutritional Support assessment	Mini Nutritional Assessment or Subjective Global Assessment Protein optimization, micronutrient supplementation Enteral or parenteral nutrition
Physical Rehabilitation	Resistance and strength training Physical therapy and mobility support
Management of Comorbidities	Medications, lifestyle modification, monitoring
Psychosocial and Cognitive Interventions	Mental health support Pharmacologic treatment when indicated Social support
Surgical Considerations	Prehabilitation programs Postoperative rehabilitation Early mobilization
Multidisciplinary and Integrated Care	Personalized care plans Continuous reassessment
Pharmacologic approaches targeting sarcopenia	Selective androgen receptor modulators or myostatin inhibitors
Telemedicine and remote monitoring	Early detection of frailty-related complications, supportexercise adherence, improve patient engagement
Preventive strategies	Vaccination, infection prophylaxis, fall prevention

## Data Availability

No new data were created or analyzed in this article.
